# Extrusion enhances apparent metabolizable energy, ileal protein and amino acid digestibility of palm kernel cake in broilers

**DOI:** 10.5713/ajas.19.0964

**Published:** 2020-03-12

**Authors:** Hanim Shakirin Faridah, Yong Meng Goh, Mohamed Mustapha Noordin, Juan Boo Liang

**Affiliations:** 1 Institute of Tropical Agriculture and Food Security, Universiti Putra Malaysia, 43400 UPM Serdang, Selangor, Malaysia; 2 Faculty of Biotechnology and Biomolecular Sciences, Universiti Putra Malaysia, 43400 UPM Serdang, Selangor, Malaysia; 3 Department of Veterinary Pre Clinical Science, Faculty of Veterinary Medicine, Universiti Putra Malaysia, 43400 UPM, Serdang, Selangor, Malaysia; 4 Department of Veterinary Pathology & Microbiology, Faculty of Veterinary Medicine, Universiti Putra Malaysia, 43400 UPM, Serdang, Selangor, Malaysia

**Keywords:** Palm Kernel Cake, Physical Treatment, Sieving, Extrusion, Hydration Property, Feedstuff

## Abstract

**Objective:**

This study consisted of two stages; the first was to determine the effect of extrusion and sieving treatments on the chemical properties of palm kernel cake (PKC), and accordingly, a follow-up experiment (second stage) was conducted to determine and compare the apparent metabolizable energy (AME), and protein and amino acid digestibility of extruded and sieved PKC.

**Methods:**

Two physical treatments, namely extrusion (using temperature profiles of 90°C/100°C/100°C, 90°C/100°C/110°C, and 90°C/100°C/120°C) and sieving (to 8 particles sizes ranging from >8.00 to 0.15 mm) were carried out to determine their effects on chemical properties, primarily crude protein (CP) and fiber contents of PKC. Based on the results from the above study, PKC that extruded with temperature profile 90/100/110°C and of sieved size between 1.5 to 0.15 mm (which made up of near 60% of total samples) were used to determine treatments effect on AME and CP and amino acid digestibility. The second stage experiment was conducted using 64 male Cobb 500 chickens randomly assigned to 16 cages (4 cages [or replicates] per treatment) to the following four dietary groups: i) basal (control) diet, ii) basal diet containing 20% untreated PKC, iii) basal diet containing 20% extruded PKC (EPKC), and iv) basal diet containing 20% sieved PKC (SPKC).

**Results:**

Extrusion and sieving had no effect on CP and ash contents of PKC, however, both treatments reduced (p<0.05) crude fiber by 21% and 19%, respectively. Overall, extrusion and sieving reduced content of most of the amino acids except for aspartate, glutamate, alanine and lysine which increased, while serine, cysteine and tryptophan remained unchanged. Extrusion resulted in 6% increase (p<0.05) in AME and enhanced CP digestibility (p<0.05) by 32%, as compared to the untreated PKC while sieving had no effect on AME but improved CP digestibility by 39% which was not significantly different from that by extrusion.

**Conclusion:**

Extrusion is more effective than sieving and serves as a practical method to enhance AME and digestibility of CP and several amino acids in broiler chickens.

## INTRODUCTION

The rising cost of conventional feedstuffs such as soybean and corn, has forced poultry producers to seek locally available alternative feedstuffs, among which palm kernel cake (PKC) is one which received much attention. Chemical composition of PKC varied, depending on the species of the palm fruit, oil extraction procedures and shell residue after the oil extraction. The high non-starch polysaccharides (NSPs) are the primary limiting factor for PKC to be used at higher inclusion rate in poultry diets. Attempts in using chemical and biological [1] treatments to increase the nutritional values of PKC have been employed with limited success [2,3]. One of the major disadvantages of biological treatment of PKC is the need to incorporate up to 50% to 60% water in the substrate to achieve optimal enhancement, which is costly to dry after treatment, particularly for large scale production. Physical treatments such as sieving, grinding and extrusion are much simpler to apply and have shown that they could influence the nutritive value of feed [3] and thus the efficiency of nutrient utilization in animals.

The primary objective of sieving and grinding is the reduction of particle size which increases the surface area per unit volume, allowing greater access to digestive enzymes to increase nutrients digestibility and utilization in animals. In addition, reduction in particle size of feed is also reported to enhance apparent metabolizable energy (AME) of broiler diet [4]. Extrusion, on the other hand, is a technology employing a high temperature for a short period of time to produce wide variety of foods and feed ingredients. The exposure of heat can also degrade microorganism and heat labile anti-nutrients. The high shear forces can break the protein structure and thus improving digestibility and offers a low-cost and versatile treatment option to improve nutrient utilization of fibrous feed. Although extrusion has been shown to improve nutrient availability, extreme temperature or pressure may destroy or alter the physical and/or chemical structures of the treated materials to become more resistant to digestion and thus becomes detrimental to animal performance [5].

Physical treatments, including extrusion and sieving provide alternatives to enzyme treatment. Extrusion has been successfully used to breakdown the fiber component of sorghum grain and therefore effectively enhances the utilization of the bonded carbohydrate and protein components resulting in a significant weight gain enhancement in growing sheep [6]. Similarly, Ahmed and co-workers [7] reported that extrusion enhanced metabolizable energy and amino acid digestibility of canola seeds in poultry. In addition, extrusion increased contents of starch and reduced sugars while sieving enhanced availability of soluble protein [8]. We do not know of any report on the comparative advantages of extrusion and sieving on fibrous feedstuffs, particularly PKC, in poultry diets. Thus the objectives of this study were i) to determine the effect of the above two physical treatments, namely extrusion and sieving under different conditions, on the chemical and nutrient composition of PKC, and ii) to compare the enhancement of the most appropriately treated PKC using extrusion and sieving on AME, protein and amino acid digestibility in broiler chickens.

## MATERIALS AND METHODS

### Animal care

The study was carried out in compliance with the principles of animal welfare and rights approved by the Universiti Putra Malaysia Research Policy for Animal studies.

### Preparation of palm kernel cake

The PKC used for this study was purchased from a local palm kernel oil extraction mill (Ace Edible Oil Industries, Malaysia). The PKC was packed in plastic bags and transported to the laboratory and stored at 4°C before use. The chemical composition of the PKC is presented in Table 1. However, its shell content was not determined but is estimated to be in the region of about 7% [9]. Extrusion of PKC was carried out using a laboratory scale stand-alone single-screw extruder with a throughput of 2 kg/h (Brabender KE19; Brabender GmbH, Duisburg, Germany). The extruder had a barrel length of 42 cm and barrel diameter of 19 mm, with a length to diameter ratio of 22:1. A uniform pitch screw with a length to diameter ratio of 25 was used in the treatment. The maximum screw torque was 150 Nm and the compression ratio achieved inside the barrel was 3:1. The barrel which consisted of three zones was heated electrically with a heating and cooling jacket. Three extrusion temperature profiles: namely 90°C/100°C/100°C; 90°C/100°C/110°C, and 90°C/100°C/120°C were pre-tested to determine the optimal condition. Water was added to the PKC to achieve a moisture content of 50% before feeding the PKC to the extruder which was operated at a preset feeder, screw and blade cutter speed of 150 rpm. Based on the pre-run results, extrusion with temperature profile of 90°C/100°C/110°C protocol which resulted in the lowest crude fiber (CF) content was used to produce the extruded PKC (EPKC) of subsequent studies (soluble protein, reducing sugars, hydration properties, AME and ileal protein and amino acid digestibility). The extrudates were oven-dried at 45°C for three days and stored at room temperature for subsequent use.

Sieved PKC was prepared using mechanical sieving to separate the PKC into different particle sizes. Approximately, 500 g of PKC were used for each batch of sieving by a mechanical sieving vibrator (UTS Unit Test, 240V, Endecotts, London, UK) for 20 min at 50 Hz using BSS 410 sieves with the following nominal apertures: 8.00, 4.75, 2.80, 2.00, 1.00, 0.50, and 0.30 mm. These screen sizes were selected to give a broad spectrum of particle size ranging from >8.00 mm to 0.15 mm. The set of sieves was arranged serially in a stack with the smallest sieve at the bottom and the largest sieve at the top. Accordingly, the average particle sizes obtained were >8.00, 6.38, 3.78, 2.40, 1.50, 0.75, 0.40, and 0.15 mm. The different fractions of PKC were oven-dried at 65°C until a constant weight was achieved, cooled and kept at to room temperature (28°C). Based on the chemical composition, especially reduction in CF content of the different factions, particles with sieved size between 1.5 to 0.15 mm, which made up of approximately 60% in weight of the total sample, were used for the subsequent studies. The above sieving process was repeated to obtain sufficient material which was then vacuum-packed and stored for subsequent use.

### Gross energy, soluble protein and reducing sugars

Gross energy (GE) of the treated and untreated PKC was determined by using Isoperibol Bomb Calorimeter (Parr Instrument Co., model 1261, Moline, IL, USA) according to the standard operating instructions (No 242M).

Soluble protein content was determined according to the method of Kupski et al [10]. Protein extraction was performed in alkaline medium (pH 9.5), adding NaOH 0.02 M in the proportion 1:7 (w/v) and further homogenization in orbital shaker for 30 min. The solution was filtered with glass wool and centrifuged at 4°C and 2,240×*g* for 20 min. the supernatant was taken and the pH was adjusted to 4.5 (isoelectric point) with HCl 1 M [11] and the precipitate was dissolved in NaOH (0.02 M). The protein fractions were quantified by method described by Lowry et al [12] using an albumin curve.

Total starch content was determined using the method of AOAC (Official Method 996.11) and AACC (Method 76.13) [13]. Briefly, the chemical methods were based on the acid starch hydrolysis, then on the volumetric or gravimetric determination. The procedure was using thermostable alpha-amylase, amyloglucosidase and glucose oxidase-peroxidase buffer.

Monosaccharides concentrations were determined by high performance liquid chromatography (HPLC) (Waters, 2690, Milford, MA, USA), using a COSMOSIL Sugar-D column (4.6 mm ID×250 mm) (Nacalai, San Diego, CA, USA). The monosaccharides concentrations were assayed using an acetonitrile/water mixture (80/20; v/v) with a flow rate of 1.0 mL/min. Different concentrations of monosaccharides (glucose, fructose, glucose, and mannose) (Sigma-Aldrich, St. Louis, MO, USA) were used as standards.

### Hydration properties

The swelling capacity and water retention capacity were determined according to the method described by Robertson et al [14]. For swelling capacity, 100 mg dry PKC were hydrated in 10 mL distilled water in a calibrated cylinder (1.5 cm diameter) at room temperature. After equilibration (18 h), the bed volume was recorded and expressed as volume/g original substrate dry weight. To determine water retention capacity, 1 g of dried PKC was hydrated in 30 mL distilled water in a centrifuge tube at room temperature. After equilibrium (18 h), samples were centrifuged (3,000×*g*, 20 min). The supernatant was decanted, and the residues were weighed.

### Animal management and dietary treatments for apparent metabolizable energy and protein digestibility studies

A total of 80 one-day-old male broiler chicks (Cobb 500) were obtained from a local hatchery and raised in groups in battery cages with wire floors in a conventional open-sided house with cyclic temperatures (maximum 34°C and minimum 24°C). Chicks were fed a commercial broiler starter diet (23% crude protein [CP], 12.76 MJ/kg) from day 1 to day 21. Feed and clean drinking water were offered *ad libitum*. On day 21, 64 birds of uniform weight were selected from the pool and randomly assigned in equal numbers (n = 4) to 16 cages and offered commercial broiler finisher diet (18% CP, 13.37 MJ/kg) for the following 6 days. On day 27, the birds were fasted for 24 h to empty the intestinal contents from previous feed. On day 28 onward, the birds were offered (unrestricted) the following experimental diets (4 cages [replicates] per diet): i) basal (control) diet, ii) basal diet containing 20% untreated PKC, iii) basal diet containing 20% EPKC and iv) basal diet containing 20% sieved PKC (SPKC) to determine the AME and ileal CP and amino acid digestibility. The three treatment diets were formulated by replacing corn and soybean meal with 20% of untreated, extruded or sieved PKC (isocaloric and isonitrogenous) to meet the NRC nutrient requirements for poultry [15]. Titanium dioxide (0.3%) was included as digestibility marker.

### Samples collection

On day 32, all the birds were killed and the contents of the lower half of the ileum (defined as that portion of the small intestine extending from the Meckel’s diverticulum to a point approximately 40 mm proximal to the ileo-cecal junction) were collected. The ileum was then divided into two halves and the digesta was collected from the lower half towards the ileo-cecal junction. Ileal digesta were processed as previously described [16]. The diet (basal diet, raw PKC diet, EPKC diet, and SPKC diet) and digesta samples were analyzed for their DM, titanium oxide, CP, and amino acid contents.

### Chemical analysis and determination of amino acids

Dry matter and CP were determined according to the procedures of AOAC 2005 [12] GE. The GE was measured with an adiabatic oxygen bomb calorimeter (Parr Adiabatic Calorimeter, Parr Instrument Co., USA). Amino acid concentrations in the diet and ileal digesta were determined by HPLC [17] and following pre-column derivatization with AQC reagent (6-aminoquinolyl-Nhydroxysuc-cinimidyl carbamate, Waters, USA). Cysteine and methionine were analyzed as cysteic acid and methionine sulfone by oxidation with performic acid for 16 h at 0°C and neutralization with hydrobromic acid before hydrolysis. Tryptophan contents were determined following alkaline hydrolysis of the sample with 4.3 N LiOH·H_2_O for 16 h at 120°C and neutralization with 6 N HCl. The rest of the amino acids were hydrolyzed by 5 mL 6 N HCl for 24 h at 110°C. Titanium dioxide was determined according to procedures described [18].

### Calculation for apparent metabolizable energy, digestibility of protein and amino acids

The AME of diets was determined using the following formula on a DM basis [19].

$$\begin{array}{l} {\text{AME}}_{\text{diet}}\;(\text{MJ}/\text{kg diet})=\text{GE diet}-[\text{GE digesta}\times ({\text{TiO}}_{2}\;\text{diet}/{\text{TiO}}_{2}\;\text{digesta})].\\ \text{Digestibility }(\%)=100-[({\text{TiO}}_{2}\;\text{diet}/{\text{TiO}}_{2}\;\text{digesta})\times (\text{nutrient digesta}/\text{nutrient diet})\times 100]. \end{array}$$

The AME values of PKC were calculated [20] as follows:

$${\text{AME}}_{\text{PKC}}\;(\text{MJ}/\text{kg})=[{\text{AME}}_{\text{PKC diet}}-({\text{AME}}_{\text{basal diet}}\times 0.8)]/0.20$$

Ileal amino acid digestibility was calculated using the method described by Kadim and Moughan [21].

### Statistical analysis

Data sets were checked for their conformance to normality using the Shapiro Wilk’s test. Assessment of the conformance to normality was also based on the descriptive statistic of parameters considered in this experiment such as mean, standard error of the mean (SEM), kurtosis, skewness and frequency distribution. All values are expressed as SEM. Comparison of treatments effects was performed using one way analysis of variance, with untreated and EPKC as the treatment factor, using the general linear model procedure of SAS software (SAS Institute Inc., Cary, NC, USA). All statistical analyses were conducted at 95% confidence level.

## RESULTS

Generally, extrusion and sieving had no effect on CP and ash contents of PKC, averaged 17.6% and 5.6%, respectively. However, extrusion and sieving reduced (p<0.05) CF by 21% and 19%, respectively. Overall, extrusion and sieving reduced the content of most amino acids, except for Asp, Glu, Ala, and Lys which increased, while Ser, Cys, and Try remained unchanged (Table 1). Both physical treatments enhanced the soluble protein content (p<0.05), with sieving being more effective which increased the soluble protein content by 52%, followed by extrusion (33%) as compared to the untreated PKC. However, none of the physical treatments altered GE content of PKC, averaged 18.01 MJ/kg (Table 2). Extrusion increased starch content of PKC by nearly five folds (p<0.05) compared to the untreated PKC (155.3 vs 33.3 mg/g sample) while sieving only enhanced starch content marginally (p> 0.05). Similarly, extrusion increased total reducing sugar content 8.5 folds (754.7 vs 88.7 mg/g sample) as compared to the untreated PKC (p<0.05) while sieving did not affect reducing sugar as compared to the untreated PKC. Extrusion also increased glucose and fructose contents (p<0.05) while contents of the above two sugars were not affected by sieving. Except for extrusion, xylose and mannose were not detected in the untreated and the sieved samples.

The effects of different physical treatments on the hydration properties of treated and untreated PKC are shown in Table 3. Only sieving increased swelling capacity (28%) and water retention capacity (16%) while extrusion did not affect the above two hydration properties as compared to the untreated PKC, averaged 2.50 mL/g and 3.07 g/g for swelling and water retention capacity, respectively.

Based on the amino acid content of the untreated and treated PKC, the feed ingredient composition (Table 4) and nutrient and amino acid composition (Table 5) of the different experimental diets used for the AME and amino acid digestibility studies were calculated. The four diets were isocaloric (13.37 MJ/kg) and isonitrogenous (18.8% CP), except that the CF of diets containing PKC was higher than that of the basal diet. In general, contents of all amino acids were higher in diets containing PKC except for lysine and methionine which were similar in all diets.

Results of the effects of the physical treatments on AME, CP, and amino acid digestibility are shown in Table 6. Extrusion resulted in a 6% increased (p<0.05) in AME, while sieving did not alter the AME of PKC as compared to the untreated PKC. However, sieving increased CP digestibility by 40% while extrusion enhanced CP digestibility by 32%. There was no difference in CP digestibility between the two treatments. As for amino acid digestibility extrusion resulted in no significant change of essential amino acids such as methionine, lysine, isoleucine, and tryptophan. Similar effects were observed for sieving.

## DISCUSSION

### Effect on chemical composition of palm kernel cake

Results of the present study showed that CP content of PKC was not affected by sieving and extrusion. This result was unexpected because sieving, which was thought to be a practical method of removing contaminants, particularly the residual shell content, and therefore, would improve the CP content of PKC. Similarly, extrusion also did not enhance the CP content of PKC. Amino acid content is a more realistic measure of protein quality because CP values usually include compounds such as nucleic acid, which have no known nutritional value. Among the sulfur-containing amino acids determined for PKC in the present study, methionine was slightly higher while cysteine content was slightly lower than the previously reported [22]. The highest amino acid content recorded in PKC was glutamic acid (1.69%). Overall, extrusion and sieving reduced most of the amino acid content except for aspartate, glutamate, alanine and lysine which increased, while serine, cysteine and tryptophan remained unchanged. The reduction of amino acid content in EPKC could be explained by Mailard reaction occurring during extrusion [23]. During extrusion, free amino acids could be produced due to heat processing in the barrel, and the free amino acids could then react with reducing sugars or other compounds present, resulting in Mailard production [24] and leading to reduced amino acids available. On the other hand, the lower amino acid content in sieved PKC as compared to the untreated one could be resulted from selection of the 1.50 to 0.15 mm particle sizes of the sieved PKC for this study.

On the other hand, CF content of sieved and EPKC reduced by 19% and 23%, respectively, as compared with the untreated PKC. The reduction of CF content of EPKC could be due to thermal decomposition [25] as well as the shear force produced by the high screw speed and temperature which caused breakage of the chemical bonds, creating smaller particles which are more soluble. There is no previous report on the effect of extrusion on CF content of PKC but treatment using cellulolytic enzymes was reported to reduce CF in PKC by 39% [26]. The nearly two folds reduction in CF content of PKC by enzyme treatment as compared with extrusion is not surprising because the former method used enzymes specifically to hydrolyze the PKC fiber while extrusion merely used mechanical force to break the fiber component. However, enzymatic treatment as reported above has a drawback because enzyme treatment requires 60% moisture content in the substrate to achieve the desired effect which results in high cost to dry the treated PKC in large scale production. As for other feed ingredients, extrusion reduced the total and insoluble dietary fiber contents in quinoa, a pseudo-cereal used as human food [27]. Ahmed et al [7] also reported that extrusion reduced CF content of canola meal by 21%. However, extrusion had no effect on dietary fiber of durum wheat [28]. The inconsistency among reports could be due to variability in the raw materials and the different experimental conditions, including screw speed, feed moisture and barrel pressure and temperature during extrusion.

Crude fat content of sieved PKC did not differ with, but extrusion increased crude fat by 20% as compared to the untreated PKC. However, the increased crude fat did not increase the GE content of the EPKC (Table 2). Neither sieving nor extrusion altered the ash content of PKC.

### Effect on hydration properties of palm kernel cake

Understanding of effect of physical treatments on hydration properties of fibrous materials is important to better understand the behavior of fiber in foods or during gut transit. High swelling capacity (the volume occupied by a known weight of sample under gravity forces) indicates increased surface area resulting in higher ability to trap more water. Results of the present study showed that only sieving increased (p<0.05) swelling capacity and water retention capacity as compared to the untreated and those treated by extrusion (Table 3). The higher swelling capacity could be attributed to presence of higher water soluble materials [14], increased surface area, total pore volume and other structural modification [4,29].

Extrusion involves shear forces with high temperature and thus is expected to alter the nutritional value such as the fiber content, starch gelatinization and hydration properties of a feed material. Surprisingly, our study showed that extrusion had no significant impact on hydration properties of PKC despite the significant (p<0.05) reduction in CF content of PKC (Table 1) and enhancement in soluble protein, starch and reducing sugars (Table 2). In other words, our results suggested that fiber composition is not the sole factor influencing water uptake in PKC, which is in agreement with a previous report [30] that fiber composition of feedstuff was not responsible for the changes in hydration properties.

The swelling capacity of PKC in this study was slightly higher than previously reported [31]. The discrepancy could be attributed to different PKC sources and physical characteristics (such as uneven surface area, and inconsistent particle shape) of PKC [32]. When compared to other feed ingredients, the swelling capacity of PKC was lower than that of wheat bran, oat bran, coconut residue and artichoke flour (ranging from 5.0 to 84.0 mL/g) [29]. Water retention capacity is the amount (g) of water retained per g dry sample, subject to a centrifugal force [33]. As stated earlier, only sieving increase water retention capacity as compared to the untreated PKC and those treated by extrusion.

### Effect on soluble protein, starch and reducing sugar

The primary objective of physical treatments of feed ingredients including agricultural products is to breakdown their fiber component to release the bonded nutrients, making them more readily available to the animals. Results of our study showed that sieving and extrusion increased soluble protein in PKC with sieving been more effective than extrusion. However, extrusion enhanced the release of starch (by nearly 4 folds) and reducing sugars (by 7 folds) as compared to the untreated and those by sieving. The latter did not differ with the untreated PKC. Similarly, extrusion also enhanced the release of glucose and fructose while sieving had no such effect. Thus, based on the present study, extrusion seems to be the more promising among the two physical treatments tested. Physical treatments have the advantages of versatility, low cost, safe from bacterial contamination, and easy to handle [20]. However, further evaluation on the effect of the above two treatments on the efficiency of utilization of the treated PKC is required to determine their actual usefulness.

### Effect on apparent metabolizable energy

The AME value of the untreated PKC determined in the present study (13.21 MJ/kg) is relatively high, being close to traditional feed ingredients such as corn (14.02 MJ/kg) and soybean (13.81 MJ/kg) and higher than barley (11.04 MJ/kg). It was reported that ME of several varieties of Chickpeas ranged from 10.25 to 13.82 MJ/kg DM [34] and that for seven bread wheat varieties ranged from11.23 to 13.17 MJ/kg [35]. We are not able to provide a reason for the high AME of the PKC obtained in this study as the fat content of the untreated PKC was only around 5.4%. However, our value is near to the TME of PKC (12.37 MJ/kg reported by Muangkeow and Chinajariyawong [36] in Thailand. In addition, the AME of the untreated PKC in this study was higher than those reported previously for PKC which ranged from 9 to 11.1 MJ/kg [37,38]. The discrepancy among the different studies is not surprising because, as previously mentioned composition, including fiber and oil contents of PKC differed from sources and the different protocols used to evaluate the AME.

Interestingly, among the two physical treatments, only extrusion enhanced AME (by 6.3%) while sieving did not improve AME as compared to the untreated PKC. Our results agreed with earlier studies which showed that extrusion of feedstuff increases AME [7]. We, however, hypothesize that in addition to the lower fiber content of the EPKC, the enhancement of AME in the EPKC could also be due to its higher starch and reducing sugars content as compared to the untreated and PKC treated by sieving.

On the other hand, the negligible effect of sieving on AME of PKC is not surprising because sieving is merely a process to separate the material into different particle sizes without altering the physical and chemical structures. Perhaps, the use of particle size of 1.5 to 0.15 mm in this study could also had some influence on the results. This argument is supported by study of Svihus et al [39] which showed that particle size of wheat had no effect on the AME. Similarly, Kim et al [40] also reported that sieved maize dry distillers grains with solubles did not improve energy digestibility. However, Esposito et al [28], and Randall and Drew [41] showed that small particle size of maize increased the true metabolizable energy values in broiler diet.

### Effect on ileal protein digestibility

In contrast to the effect on AME, sieving resulted in higher enhancement in ileal protein digestibility by 39% while extrusion by 32% as compared to the untreated PKC. However, ileal protein digestibility among the two physical treatments were not statistically different. The higher ileal protein digestibility in the sieved PKC-diet could be due to the small particle size of the sieved PKC which have higher surface area, allowing greater access to digestive enzymes [42]. The above suggestion is reflected by the higher soluble protein content in the sieved PKC as compared to the untreated PKC or that treated by extrusion. This observation has also been reported [43] that fine particles of peas improved apparent ileal protein digestibility. It has been documented [44] that feeding small particles of wheat-based diet resulted in higher starch digestibility. Increased protein digestibility by extrusion of PKC could be due to the exposure to high temperature and pressure which breakdown the protein bind to the fiber component of the PKC allowing better enzyme to digest the protein molecules [45]. Similar observation was reported of higher protein digestibility when extruded canola meal was fed to broiler chicken [7].

### Effect on amino acid digestibility

The reduction of certain amino acid digestibility in sieved PKC diet could be due to the influence of digesta viscosity. The NSPs present in PKC could increase the viscosity of the digesta and prolong small intestine transit time and rate of absorption of nutrients thus affecting the release of peptide which reduces the amino acid absorption [4]. There is no known study on the effect of extrusion of PKC on amino acid digestibility, but studies had shown the beneficial effects of extrusion of faba beans on amino acid digestibility [20,46].

The reduction of tyrosine and phenylalanine digestibility in the EPKC (Table 5) could be due to the presence of hydrophobic residue in the tyrosine and phenylalanine structure which make them less soluble thus reduce digestibility. During extrusion, the native protein denatured, weakening the protein structure and allowing tyrosine and phenylalanine to align with the flow material towards the die. The process of extrusion exposed the hidden amino acids residues, and freed them to react with reducing sugars and other food components. This action, increased the amino acid solubility [47], thus increasing the amino acid digestibility. The higher of protein digestibility compared to amino acid digestibility in treated PKC could be explained by the CP measurement that measures all nitrogen content from protein sources and non protein sources.

## CONCLUSION

Although several studies have shown that enzyme treatment significantly reduces fiber content of PKC but the efficacy of the enzyme treated PKC to enhance growth performance in chicken is below expectation. An additional drawback of enzyme treatment is the high cost of drying the treated PKC in large scale production. Physical treatments such as extrusion and sieving may provide cost effective and practical alternatives to biological treatments to enhance the nutritive value of PKC as poultry feed. Our results showed that extrusion and sieving increased protein digestibility by 30%, however, only extrusion enhanced AME of PKC in broiler chickens. Thus, extrusion is preferred over sieving in enhancing AME of PKC as feed ingredient for broiler chickens.

## Figures and Tables

**Table 1:** Effect of extrusion and sieving on chemical composition of palm kernel cake

Items	Untreated PKC	Extruded PKC	Sieved PKC	SEM
Analyzed composition (%)				
Dry matter	93.4	95.9	93.9	0.59
Crude protein	17.6	17.3	17.9	0.21
Crude fiber	18.3^a^	14.4^b^	14.9^b^	0.20
Crude fat	5.4	6.2	5.5	0.04
Ash	4.4	4.6	5.4	0.06
Amino acids (g/100 g)				
Asp	0.73^b^	1.12^a^	1.39^a^	0.04
Ser	0.79	0.79	0.78	0.04
Glu	1.69^b^	2.38^a^	2.89^a^	0.02
Gly	0.83^a^	0.62^b^	0.73^b^	0.01
His	0.33^a^	0.18^b^	0.22^b^	0.01
Arg	2.35^a^	1.64^b^	1.64^b^	0.02
Thr	0.57^a^	0.45^b^	0.38^b^	0.01
Ala	0.52^b^	0.62^a^	0.59^a^	0.01
Pro	0.68^a^	0.57^b^	0.62^a^	0.00
Tyr	0.92^a^	0.38^b^	0.44^b^	0.01
Val	0.61^a^	0.54^b^	0.56^b^	0.03
Met	0.39^b^	0.30^b^	0.62^a^	0.01
Lys	0.42^b^	0.54^a^	0.47^b^	0.02
Iso	0.41^a^	0.38^a^	0.57^b^	0.01
Leu	1.20^a^	1.01^b^	0.92^b^	0.01
Phe	1.50^a^	0.65^b^	0.74^b^	0.03
Cys	0.05	0.04	0.04	0.00
Trp	0.12	0.13	0.11	0.04

Values were based on triplicate measurement.

PKC, palm kernel cake; SEM, standard error of the mean.

a,b Values within a row with different superscripts differ significantly (p<0.05).

**Table 2:** Soluble protein, gross energy, starch and reducing sugars of untreated palm kernel cake and those treated by the different physical treatments

Nutrient (% per DM)	Untreated PKC	Extrusion	Sieving	SEM
Soluble protein (mg/g per DM)	2.04^c^	2.72^b^	3.09^a^	0.20
Gross energy (MJ/kg)	17.98	17.96	18.10	0.20
Starch (mg/g DM)	33.29^b^	155.3^a^	44.00^b^	0.76
Reducing sugar (mg/g sample)	88.66^b^	754.70^a^	86.55^b^	0.04
Glucose (mg/g sample)	19.69^b^	26.16^a^	9.25^c^	0.10
Fructose (mg/g sample)	19.73^b^	26.24^a^	7.56^c^	0.04
Xylose (mg/g sample)	ND	5.01	ND	0.01
Mannose (mg/g sample)	ND	7.47	ND	0.03

Values were based on triplicate measurement.

DM, dry matter; PKC, palm kernel cake; SEM, standard error of the mean; ND, not detected.

a–c Values within a row with different superscripts differ significantly (p<0.05).

**Table 3:** Hydration properties of the untreated and treated PKC by various physical treatments

PKC treatment	Swelling capacity (mL/g)	Water retention capacity (g/g)	SEM
Untreated	2.53^b^	2.98^b^	0.01
Extrusion	2.49^b^	2.93^b^	0.01
Sieving	3.25^a^	3.45^a^	0.05

Values were based on triplicate measurement.

PKC, palm kernel cake; SEM, standard error of the mean.

a,b Values within a column with different superscripts differ significantly (p<0.05).

**Table 4:** Feed and calculated composition of diets (%) for AME and protein and amino acid digestibility studies

Feed composition of diets (%)	Basal diet	Untreated PKC diet	Extruded PKC diet	Sieved PKC diet
Ingredient				
Corn grain	61.43	46.40	46.45	48.26
Soybean meal (CP, 48%)	29.20	24.40	24.85	24.85
Raw PKC	0.00	20.00	0.00	0.00
Extruded PKC	0.00	0.00	20.00	0.00
Sieved PKC	0.00	0.00	0.00	20.00
Palm oil	5.50	5.50	5.00	5.00
Dical. Phos.	1.60	1.30	1.30	1.30
Limestone	1.05	1.15	1.15	1.15
Vitamin premix1)	0.08	0.08	0.08	0.08
Mineral premix2)	0.05	0.05	0.05	0.05
DL-methionine	0.20	0.18	0.18	0.19
L-lysine HCl	0.23	0.28	0.28	0.32
Choline Cl, 70%	0.00	0.00	0.00	0.00
Common salt	0.36	0.36	0.36	0.36
Titanium dioxide	0.30	0.30	0.30	0.30
Calculated composition of diets (%)				
Dry matter (%)	88.99	90.14	90.89	89.79
Metabolizable energy (MJ/kg)	13.37	13.37	13.37	13.37
CP (%)	18.81	18.81	18.82	18.82
Ether extract (%)	8.23	8.57	8.08	8.04
Linoleic acid	1.85	1.52	1.48	1.51
Crude fiber	3.64	6.31	5.35	5.87
Calcium	0.86	0.85	0.85	0.85
Total phosphorus	0.74	0.74	0.74	0.74
Total amino acids				
Arg	1.24	3.55	3.57	3.52
Gly	0.77	1.53	1.54	1.51
Ser	0.92	1.61	1.62	1.59
His	0.52	0.84	0.85	0.83
Ile	0.78	1.34	1.35	1.32
Leu	1.59	2.52	2.54	2.49
Lys	1.06	1.06	1.07	1.06
Met	0.46	0.46	0.46	0.46
Cys	0.14	0.35	0.36	0.35
Phe	0.35	1.06	1.07	1.06
Tyr	0.48	0.90	0.90	0.88
Thr	0.72	1.21	1.21	1.19
Trp	0.20	0.31	0.31	0.30
Val	0.93	1.76	1.77	1.74

PKC, palm kernel cake; CP, crude protein.

1) Supplied per kg of the diet: vitamin A (retinyl acetate), 8,000 IU; vitamin D (cholecalciferol), 1,000 IU; vitamin E (DL-α-tocopherol), 30 IU; vitamin K_3_ (menadione dimetylpyrimidinol), 2.5 mg; vitamin B_2_, 5 mg; vitamin B_6_, 2 mg; vitamin B_12_, 0.01 mg; niacin, 30 mg; d-biotin, 0.045 mg; vitamin C, 50 mg; d-pantothenate, 8 mg, folic acid, 0.5 mg.

2) Supplied per kilogram of the diet: Mn, 70 mg; Fe, 35 mg; Zn 70 mg; Cu, 8 mg; I, 1 mg; Se, 0.25 mg; Co, 0.2 mg.

L-Lysine: lysine, methionine, threonine, and tryptophan were feed grade, while the rest of supplemented amino acids were pharmaceutical grade.

**Table 5:** Apparent metabolizable energy and ileal crude protein and amino acids digestibility of untreated and treated palm kernel cake in broiler chickens.

Items	Untreated PKC	Extruded PKC	Sieved PKC	SEM
AME (MJ/kg DM)	13.2^b^	14.0^a^	13.4^b^	1.2
CP digestibility (% DM)	70.0^b^	80.5^a^	85.2^a^	0.7
Amino acids digestibility				
Glu	78.8	75.7	81.0	0.0
Gly	77.1^a^	69.3^b^	75.6^a^	0.3
Thr	74.6^a^	62.6^b^	70.7^ab^	0.3
His	85.6^a^	65.7^b^	77.5^b^	0.3
Pro	76.9^a^	66.4^b^	75.6^a^	0.2
Arg	86.2^a^	72.9^b^	81.7^ab^	0.0
Ile	78.5	73.9	75.8	0.1
Ala	94.9	93.5	94.3	0.6
Cys	69.4	63.9	67.1	0.0
Met	62.4	66.6	66.9	0.3
Val	76.8^a^	65.9^b^	78.0^a^	0.3
Lys	78.1	76.39	84.9	0.2
Asp	67.8^b^	71.6^a^	75.9^a^	0.0
Tyr	87.4^a^	60.7^b^	70.9^b^	0.1
Ser	80.5^a^	68.2^b^	77.9^ab^	0.2
Leu	81.1^a^	67.7^b^	78.2^a^	0.3
Phe	88.3^a^	60.7^b^	76.3^b^	0.2

PKC, palm kernel cake; SEM, standard error of the mean; AME, apparent metabolizable energy; CP, crude protein; DM, dry matter.

Values were based on triplicate measurement.

a,b Values within a row with different superscripts differ significantly (p<0.05).
